# High COVID-19 Vaccine Acceptance among Eye Healthcare Workers in Uganda

**DOI:** 10.3390/vaccines10040609

**Published:** 2022-04-14

**Authors:** Juliet Otiti-Sengeri, Omaido Blair Andrew, Rebecca Claire Lusobya, Immaculate Atukunda, Caroline Nalukenge, Abubakar Kalinaki, John Mukisa, Damalie Nakanjako, Robert Colebunders

**Affiliations:** 1 College of Health Sciences, Makerere University, Kampala P.O. Box 7072, Uganda; bomaido@georgina.co.ug (O.B.A.); clairerebecca.lusobya@mak.ac.ug (R.C.L.); atukunda.immaculate@mak.ac.ug (I.A.); cnalukenge@georgina.co.ug (C.N.); abubakar.kalinaki@mak.ac.ug (A.K.); john.mukisa@bcm.edu (J.M.); damalie.nakanjako@mak.ac.ug (D.N.); 2 Department of Ophthalmology, School of Medicine, College of Heath Sciences, Makerere University, Kampala P.O. Box 7072, Uganda; 3 Infectious Diseases Institute, College of Health Sciences, Makerere University, Kampala P.O. Box 22418, Uganda; 4 Department Clinical Science, Institute of Tropical Medicine, 2600 Antwerp, Belgium; robert.colebunders@uantwerpen.be; 5 Global Health Institute, University of Antwerp, 2610 Antwerp, Belgium

**Keywords:** COVID-19, vaccine acceptance, vaccine hesitancy, COVID-19 vaccine uptake, healthcare workers, ophthalmology, health belief model, Uganda

## Abstract

Background: Protecting healthcare workers against COVID-19 disease is crucial, and COVID-19 vaccination is the most effective method to do so. Eye healthcare workers provide routine care in proximity, increasing infection risk, hence their need for full vaccination. This study determined COVID-19 vaccine acceptance and barriers to its uptake among eye healthcare workers practicing in Uganda. Methods: This was a cross-sectional online and telephone survey based on the health belief model (HBM), conducted in June–August 2021. A modified Poisson regression model with robust standard errors was used to determine the factors associated with COVID-19 vaccine acceptance. Results: In total, 300 (85%) of the 357 eye healthcare workers participated in the study with mean age 43 ± 8 years and 182 (60.7%) were men. Overall, 97.6% (95% CI: 95.9–99.4) had accepted and/or were willing to take the COVID-19 vaccine, 65.3% had received a shot of the COVID-19 vaccine, and 97 (32.3%) reported the intention to accept the vaccine when it became available. Among the HBM constructs, high perceived susceptibility and high perceived benefits were significantly associated with COVID-19 vaccine acceptance. Conclusions: The acceptance of the COVID-19 vaccine among eye healthcare workers in Uganda is very high. There is a dire need to make vaccines available to developing nations like Uganda.

## 1. Introduction

The corona virus disease (COVID-19) was declared a pandemic by the World Health Organization (WHO) on 11 March 2020 [1]. It is a respiratory infectious disease caused by severe acute respiratory syndrome coronavirus 2 (SARS-CoV-2). As of 16 October 2021, Uganda had registered 125,261 confirmed cases with 3185 deaths [2]. The Ministry of Health was supplied with 864,000 vaccine doses, shipped via the global COVID-19 Vaccine Access (COVAX) facility on 5 March 2020, and as of 5 May 2021, the country had vaccinated 364,582 people with the first dose of the AstraZeneca COVID-19 vaccine, and 0.89 doses per 100 people had been administered [3,4]. Uganda intends to vaccinate about 49.6% of its approximately 46 million citizens, although only 5% of the population in Uganda was vaccinated by 17 October 2021 [5].

Vaccination is the most effective strategy for stopping the pandemic and avoiding complications associated with this communicable disease [6]. However, many studies have shown that the decision to take a vaccine is dependent on beliefs and perceptions. Previous studies showed a strong relationship between the knowledge and attitudes of healthcare professionals about vaccines and their vaccine recommendations for their patients [7]. Since healthcare workers are considered the most reliable source of information on vaccines, their hesitancy about vaccination may weaken trust and consequently strongly impact on vaccine uptake in the general population.

Eye health workers are a very vulnerable group due to the proximity within which they examine their patients, greatly exposing them to patient secretions [8]. Vaccine hesitancy among eye healthcare professionals means that they can become spreaders of the virus since they can easily get infected, thereby infecting subsequent patients in their care.

Vaccine hesitancy in a growing phenomenon globally and is recognized by the WHO as a top threat to public health as shown by several studies [9]. Vaccine hesitancy as defined by WHO is the delay in acceptance or refusal of vaccines despite the availability of vaccine services. This is influenced by factors such as complacency, convenience, and confidence in the vaccine [7].

Thus, this study aimed to determine the prevalence of COVID-19 vaccine acceptance, and factors that may affect vaccination decision-making among eye health workers in Uganda using the health belief model.

## 2. Materials and Methods

### 2.1. Study Design

This was a cross-sectional study with a country-wide scope utilizing quantitative methods from 2 June 2021 to 27 August 2021.

### 2.2. Study Setting

Eye healthcare workers in Uganda are found at all levels of the health system. Most patients with ophthalmological problems are seen at the referral level due to the increased work force, availability of more specialized eye healthcare workers and equipment. These are usually at private-not-for-profit hospitals, regional, and national referral levels. There are 357 actively serving eye healthcare workers in the private-non-for profit (PNFP), regional, and national referral hospitals in Uganda at various levels of specialty from ophthalmologists (40), ophthalmologic clinical officers (OCOs) (200), optometrists (17), to nurses (100). Their contact information is accessible through their respective professional organizations, i.e., the Uganda ophthalmology society, National Association of OCOs and Cataract Surgeons, the Uganda Optometrists Organization, and the Uganda Nurse and Midwives Council.

### 2.3. Study Population

All consenting practicing eye healthcare workers in Uganda who were fully registered by their professional regulatory bodies and were actively working in the health service were recruited into this study.

### 2.4. Study Tool and Procedure

Eye healthcare workers were reached via email to fill in a self-administered questionnaire after getting informed on the objectives and activities of this study. The questionnaire and consent were hosted on Google Forms, and a link to was shared with the participants via email. For individuals without ready access to the internet or emails, two research assistants recruited these individuals by telephone, and the questionnaire was administered verbally following obtaining verbal informed consent. Independent variables were as follows: psychosocial and demographic data, which included age, gender, marital status, history of a chronic illness, occupation, perceived overall health status, experience of COVID-19 infection, training on COVID-19, years of experience, and the health belief model (HBM) constructs (perceived susceptibility, perceived severity, perceived benefits, and perceived barriers). Each of the five constructs were measured on a binary scale of Agree/Disagree. The dependent variable was the primary outcome variable—acceptability of the COVID-19 vaccine, which was assessed using a closed ended question with three responses: “Yes, I have received the COVID-19 vaccine/No, but I intend to receive the vaccine/No, I don’t intend to receive the COVID-19 vaccine”. We pretested this questionnaire among 10 non-eye healthcare workers in Uganda. To minimize the spread of COVID-19, we used a non-physical contact method, i.e., an online and telephone survey. The internal consistency of the perception towards COVID-19 vaccination by using the HBM constructs was assessed by calculating the Cronbach alpha values for each HBM concepts.

### 2.5. Data Management and Analysis

The fully completed questionnaires were extracted from Google Forms and exported to Microsoft Excel to be merged with data collected verbally. The data were cleaned, coded in Excel, and then exported to STATA 15 (Stata Corp LLC., College Station, TX, USA) for analysis. Categorical data related to demographic variables were presented as frequencies/proportions. Continuous variables are presented as mean ± standard deviation (SD) or median (inter-quartile range) depending on their distribution assessed by Kolmogorov–Smirnov test.

The HBM constructs questions were assigned +1 for agree and −1 for disagree. We then derived a sum for each of the four constructs, and those who scored more than zero were designated to have a high perception, whereas those who scored zero or below were designated to have a low perception of a given construct. This generated the categorical variable used in the regression model.

For bivariate and multivariate analysis, a modified Poisson regression with standard errors was used to determine the crude risk ratios (cRR) and adjusted risk ratios (aRR). All variables with a *p*-value of less than 0.2 were included for multivariate analysis. The interaction was assessed using the Wald test and variables with a 10% or more difference between the adjusted and crude risk ratios were considered to be confounders. Variables with *p*-values less than 0.05 at multivariable analysis were considered to be significantly associated with vaccine acceptance.

## 3. Results

In total, 301 (84%) of the 357 eye healthcare workers in Uganda consented to participate in this study. Of the 300 that completed the questionnaires, 182 (60.7%) were men and 118 (39.3%) were women, and 58 (19.3%) participated via the online survey. The average age was 43 years ranging from 24 to 66 years. The majority (78.7%) were ophthalmic clinical officers. The average number of years of experience was 13 years. Most of the participants were married (87.3%) and catholic (43.3%) by religion, with 14% reporting a chronic illness. Almost all participants (98.3%) reported perceived good health (Table 1). About half (55%) of the participants knew of acquaintances who had been infected by COVID-19. More than half of the eye healthcare workers (65.3%) had received a shot of the COVID-19 vaccine and 97 (32.3%) reported the intention to accept the vaccine when it became available.

**Figure 1:**
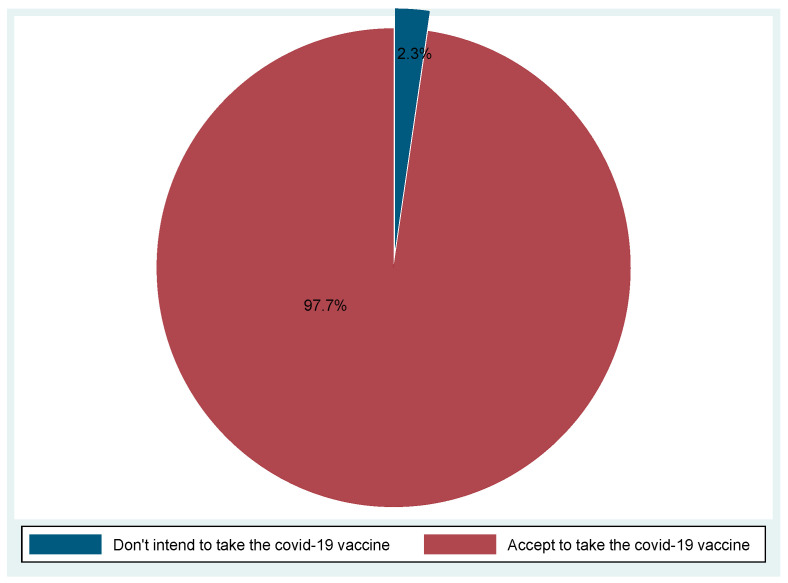
Acceptance/intention to receive COVID-19 vaccine.

The majority, 97.6% (95% CI 95.9–99.4), of the eye healthcare workers in Uganda accepted and were willing to take the COVID-19 vaccine. The prevalence of vaccine hesitancy was 2.3% (95% CI 0.6–4.1) (Figure 1).

Table 2 shows the distributions of the responses to the HBM constructs. For the perceived susceptibility, the majority (97.7%) agreed that getting infected with COVID-19 was a possibility. Among the perceived benefits of COVID-19 vaccination, most the eye healthcare workers (74.7%) agreed that the vaccination would decrease their risk of getting COVID-19 or its severe complications.

At bivariate analysis, perceived susceptibility (RR = 1.08, *p* = 0.038) was significantly associated with acceptance/intention to receive a COVID-19 vaccine, whereas perceived benefits (RR = 1.06, *p* = 0.06) was borderline significant (Table 3).

In multivariable analysis, eye health workers with high perceived susceptibility (aRR = 1.05, *p* = 0.029) were 5% more likely to accept the COVID-19 vaccine when compared to those with low perception. High perceived benefits (aRR = 1.07, *p* = 0.034) showed a similar effect at 7% increased likelihood to accept COVID-19 vaccination when compared to those with low perception (Table 4).

Concerns about the COVID-19 vaccination process are shown in Table 5. Most of the eye health workers were concerned about the limited availability of COVID-19 vaccine doses in Uganda.

## 4. Discussion

### 4.1. Prevalence of Vaccine Acceptance

Vaccine acceptance among eye health workers in Uganda was very high (97.3%). This is similar to a study among the general population in China (91% acceptance) [10]; however, it is very different from a study in the Democratic Republic of Congo (DRC), where only 27.7% of healthcare workers were willing to take the vaccine [11]. In Israel, 78% percent of the doctors were willing to be vaccinated against COVID-19 [12]. The high vaccine acceptance rate in Uganda could also be because the vaccine is distributed free of charge, unlike in some other countries where willingness to pay played a role in vaccine acceptance (13). The high acceptance rates among the eye health workers in Uganda could positively impact the vaccination rates among their patients. Our study was conducted during the second wave in Uganda, unlike other studies conducted in the earlier phases of the pandemic among medical personnel with vaccine acceptance varying from 37% to 70% [13,14]. In a survey from July to September 2020 in Western Uganda among the general population, COVID-19 vaccination acceptance was only 53.6% [15]. In another survey conducted in September and November 2020, among Ugandan healthcare workers, only 70.2% of them were willing to be COVID-19 vaccinated, and in another survey among Ugandan medical students, this percentage was only 37.3% [13,14]. In our study, more than half (55%) of the participants had friends, neighbors, or colleagues who had been infected by the COVID-19 virus, which could have skewed participants to view COVID-19 vaccination much more positively since it prevents severe disease and reduces the chances of hospitalization [16].

### 4.2. Factors Associated with Vaccine Hesitancy

Eye health workers with high perceived susceptibility were more likely to accept COVID-19 vaccination compared to those with low perceived susceptibility. This is similar to a study in Saudi Arabia and Ethiopia [17,18]. Eye health workers with high perceptions of benefits were more likely to accept COVID-19 vaccination compared to those with low perceptions of benefits. This is similar to a study among the general population in Malaysia and Hong Kong [19,20].

Age was not found to be a significant factor associated with acceptance/intention to receive the COVID-19 vaccine. This is unlike studies done elsewhere among health workers in France, the DRC, and Hong Kong [11,21,22] and in the general population where older age was found to be associated with potential acceptance of the COVID-19 vaccine [23,24]. As individuals get older, they develop a higher perception of susceptibility to develop severe disease and therefore are more likely to accept vaccination [25]. Irrespective of age, perceived risk of infection was high among health workers because of potential exposure from their patients/clients.

Sub-group analysis using Pearson’s’ chi square among participants who intended to receive and those who had already received the vaccine showed that in the two groups there was no association between vaccine acceptance and age, gender, marital status, or religion and years of professional life. However, in the two groups, there was a significant association with the following variables: “I am worried about getting COVID-19 infection” and “My risk of getting COVID-19 in the next few months is high”. Among the HBM constructs, perceived susceptibility, benefits, and barriers showed a similar significant relationship with vaccine acceptance in the two groups.

The major concern expressed by the eye healthcare workers was the limited number of vaccine doses available in the country, which is a concern not only in Uganda, but also in the most of sub-Saharan Africa. This has been partly addressed by the COVAX facility initiative, which allows for pooling resources by different African countries in order to purchase adequate numbers for their populations [26,27].

Specific concerns expressed by the seven individuals who reported that they would not take the vaccine were: “Not much information has been shared about the vaccine”, “The vaccine development process was rushed”, “No available alternative brands of vaccines”, “It should not be mandatory, let us vaccinate politicians and wait for the long-term side effect”, “We are not sure of the long-term side effect of the vaccine, no research was done on guinea pigs as for other vaccines”, “How long does it offer protection to me against the disease?”, and “We need more research on this vaccine”.

Concerns of the eye healthcare workers about the experimental nature of the vaccine could be rooted in misinformation on social media. Providing evidence-based information in appropriate media and languages understandable to all is essential and can play a role in combating the infodemic [28].

The HBM has been applied in several areas of public health, for example, to assess among ophthalmologists their risk perception, their willingness to participate in protective health behavior, and their capability to perform adequate infection prevention methods to limit the spread of coronavirus disease 2019 (COVID-19). Considering the vulnerability of the eye healthcare workers to become infected, it is vital to determine the influence of psychosocial metrics as assessed by the HBM since they have a duty to protect their families and society at large.

A limitation of this study is that we used a single item measurement to measure vaccine acceptance, yet vaccine hesitancy is complex and multidimensional. It is therefore recommended that it is measured on a Likert scale in order to exclude potential bias [29]. The study was among eye healthcare workers only, and therefore, the results are not generalizable to all healthcare workers in Uganda. This study mainly measured the intention to be vaccinated, although over 60% were already vaccinated. Future studies should measure COVID-19 vaccine completion rates, particularly in the general population where perceived risk of exposure may low.

## 5. Conclusions

This study found a high level of acceptance of COVID-19 vaccination among eye healthcare workers in Uganda at a time of limited access to vaccine doses in Uganda and much of Southern Africa. Making COVID-19 vaccines more available in sub-Saharan Africa should be a public health priority. In addition, immunization programs and communication strategies by the Ministry of Health should be designed to address lingering perceived barriers in safety and efficacy of the vaccines.

## Figures and Tables

**Table 1:** Socio demographic characteristics of eye healthcare workers in Uganda (*n* = 300).

Variable	Frequency *n* (%)
**Sex**	
Male	183 (60.8)
Female	118 (39.2)
**Age**	
≤40	112 (37.3)
>40	188 (62.7)
**Religion**	
Catholic	130 (43.3)
Muslim	33 (11.0)
Anglican	76 (25.3)
Pentecostal	55 (18.3)
Others (Atheist, *SDA)	6 (2.0)
**Marital status**	
Single	28 (9.3)
Married	262 (87.3)
Separated	8 (2.67)
Divorced	2 (0.8)
**Occupation**	
Ophthalmologist	25 (8.4)
Optometrist	22 (7.3)
Ophthalmic Clinical Officer	236 (78.7)
Nurse	1 (0.3)
Others	16 (5.3)
**History of a chronic illness**	
Yes	42 (14.0)
No	258 (86.0)
**Continuing medical education on COVID-19 and the vaccine**	
Yes	233 (77.7)
No	67 (22.3)
**Do you know of any friends, neighbors, or colleagues who are/were infected by COVID-19 virus?**	
Yes	165 (55.0)
No	135 (45.0)
**Perceived overall health**	
Very good	174 (58.2)
Good	120 (40.1)
Fair/Poor	5 (1.7)
**Have you received the COVID-19 vaccine?**	
Yes, I have	196 (65.3)
No, but I intend to	97 (32.3)
No, I don’t intend to receive the vaccine	7 (2.4)

*SDA: Seventh Day Adventist.

**Table 2:** COVID-19-related health beliefs of eye healthcare workers in Uganda (*n* = 300).

Perceived COVID-19 Health Beliefs	Agree, *n* (%)	Disagree, *n* (%)
**Perceived susceptibility**		
My risk of getting COVID-19 in the next few months is high	176 (58.7)	124 (41.3)
I’m worried about the likelihood of getting COVID-19	213 (71.0)	87 (29)
Getting COVID-19 is currently a possibility for me	293 (97.7)	7 (2.3)
Greater public awareness is needed about the COVID-19 vaccine	295 (98.3)	5 (1.7)
**Perceived severity **		
Complications of COVID-19 are serious	293 (97.7.0)	7 (2.3)
I will be very sick if I get COVID-19	258 (86.0)	42 (14.0)
I’m afraid of getting COVID-19	229 (76.3)	71 (23.7)
**Perceived benefits **		
Vaccination is a good idea because it makes me feel less worried about catching COVID-19	192 (64.0)	108 (36.0)
Vaccination will decrease my risk of getting COVID-19 or its severe complications	224 (74.7)	76 (25.3)
**Perceived barriers **		
Concerned about the efficacy of the vaccine	170 (56.7)	130 (43.3)
Concerned about the side effects/safety of the vaccine	133 (44.3)	167 (55.7)
I don’t need the vaccine because I do all the right things. I wash my hands and wear a mask and gloves	34 (11.3)	266 (88.7)
I don’t like needles	18 (6.0)	282 (94.0)

Cronbach alpha = 0.80 (HBM internal consistency was good and acceptable).

**Table 3:** Bivariable analysis of factors associated with COVID-19 vaccine acceptance among eye healthcare workers in Uganda (*n* = 300).

Variable	Acceptance and Intention to Receive COVID-19 Vaccine		cRR (95% CI)	*p*-Value
	Yes	No		
**Age**			1.01 (0.99–1.01)	0.074
**Sex**				
Male	177	5	Reference	
Female	116	2	1.01 (0.98–1.05)	0.55
**Occupation**				
Ophthalmologist	24	1	Reference	
Optometrist	21	1	1.03 (0.91–1.16)	0.681
OCO *	235	1	1.04 (0.94–1.14)	0.459
Others **	13	4	0.81 (0.62–1.06)	0.12
**Perceived overall health**				
Very good	170	4	Reference	
Good/Fair	117	3	0.97 (0.93–1.01)	0.196
**Continuing medical education on COVID-19 and the vaccine**				
Yes				
No	229	4	Reference	
	64	3	0.99 (0.96–1.03)	0.979
**Marital status**				
Married	257	5	Reference	
Not married	36	2	0.98 (0.91–1.06)	0.628
**Perceived susceptibility**				
Low	64	4	Reference	
High	229	3	1.08 (1.0–1.16)	0.038
**Perceived severity**				
Low	69	3	Reference	
High	224	4	0.98 (0.93–1.05)	0.605
**Perceived benefits**				
Low	107	6	Reference	
High	186	1	1.06 (0.99–1.12)	0.06
**Perceived barriers**				
Low	178	1	Reference	
High	115	6	1.01 (0.97–1.06)	0.555

* OCO: Ophthalmologic clinical officer. cRR: crude relative risk. ** Other occupations: ophthalmology residents (11), tutor (2), cold chain technician (1), optician (1), receptionist (1), nurse (1).

**Table 4:** Multivariable analysis of factors associated with COVID-19 vaccine acceptance among eye health workers in Uganda.

Variable	aRR (95% CI)	*p*-Value
**Age**	1.0 (0.99–1.01)	0.091
**Occupation**		
Ophthalmologist	Reference	
Optometrist	1.01 (0.91–1.13)	0.838
OCO *	1.03 (0.02–14.52)	0.458
Others	0.81 (0.01–1.36)	0.114
**Perceived overall health**		
Very good	Reference	
Good/Fair	0.97 (0.93–1.01)	0.2
**Perceived susceptibility**		
Low	Reference	
High	1.07 (1.00–1.14)	**0.034**
**Perceived benefits**		
Low	Reference	
High	1.05 (1.00–1.09)	**0.029**

* Ophthalmologic clinical officer. aRR: adjusted relative risk.

**Table 5:** Concerns about COVID-19 vaccination by eye health workers in Uganda.

Summary of Concerns Expressed by the Eye Healthcare Workers
• Doses are few for the targeted population (86 comments)
• The vaccine is experimental (33 comments)
• Inadequate information on the vaccine (23 comments)
• Long-term repercussions (21 comments)
• Vaccine is not effective against all variants (20 comments)
• A lot of misinformation on social media (1 comment)
• Guidelines are continuously changing (1 comment)
• Length of protection is unknown (1 comment)
• Time too short for development and testing (1 comment)
• No alternative ‘brands’, why AstraZeneca only (1 comment)

## Data Availability

The datasets used and/or analyzed during this study are available from the corresponding author on reasonable request.
